# How and why? Technology and practices used by university mathematics lecturers for emergency remote teaching during the COVID-19 pandemic

**DOI:** 10.1093/teamat/hrab018

**Published:** 2021-10-04

**Authors:** Eabhnat Ní Fhloinn, Olivia Fitzmaurice

**Affiliations:** 1 School of Mathematical Sciences, Dublin City University, Dublin, Ireland; 2 School of Education, University of Limerick, Limerick, Ireland

## Abstract

The COVID-19 pandemic led to closures of university campuses around the world from March 2020 onwards. With little or no time for preparation, lecturers turned to emergency remote teaching to continue to educate their students. Online mathematics education poses particular challenges in terms of both the hardware and software necessary for effective teaching, due to issues with mathematical symbols and notation, among others. In this paper, we report upon an online survey of 257 university mathematics lecturers across 29 countries, which explores what hardware and software they used for emergency remote teaching, for what purposes they used these and what training and support were made available to them at the time. We also consider what approaches they took to emergency remote teaching and what were their reasons for this.

## 1 Introduction

The first wave of the COVID-19 pandemic in late February/early March 2020 led to a series of closures of university campuses across the globe, with many lecturers turning to emergency remote teaching to continue to educate their students. While a wealth of research and experience exists in the general area of online teaching, it is important from the outset to note the difference between online teaching and emergency remote teaching, with the latter best characterized *‘as a temporary shift of instructional delivery to an alternate delivery mode due to crisis circumstances’* (Hodges *et al.*, 2020). During the initial closures of university campuses, many were mid-way through semester block of teaching and had to change their mode of delivery over the course of a weekend. Beyond the initial months of the emergency remote teaching what many lecturers have been engaged in can be described as blended (Graham, 2006), hybrid (Snart, 2010) and distance learning (Moore *et al.*, 2011), but this article is solely concerned with the emergency remote teaching that took place during the early months of the pandemic.

Teaching mathematics remotely presents specific challenges related to the nature and symbolic notation of the subject, among other challenges (Trenholm & Peschke, 2020; Glass & Sue, 2008). Therefore, the aim of this research was to investigate how and why large numbers of mathematics lecturers in higher education used particular technology to adjust to emergency remote teaching during the initial months of the COVID-19 pandemic. Specifically, this paper addresses the following research questions:

(1) What hardware and software were most commonly used by mathematics lecturers before the pandemic compared with during emergency remote teaching?
(2) Why did lecturers choose to conduct live online sessions, pre-recorded sessions or other approaches during emergency remote teaching?
(3) What training and support did staff receive in the use of hardware and software?

In order to address these research questions, an online survey was designed and distributed to a wide range of mathematics lecturers in May–June 2020 as detailed below.

## 2 Background

Research into the impact of the COVID-19 pandemic upon university education is starting to emerge, with several publications focusing on how lecturers and students reacted to emergency remote teaching, either in individual universities or regions (Bao, 2020; Bawa, 2020; Jena, 2020; Oyediran *et al.*, 2020; Rahiem, 2020) or specific subject areas, particularly in STEM fields (Alqurshi, 2020; Barton, 2020; Delgado *et al.*, 2020; Jabbar *et al.*, 2021; Tan *et al.*, 2020). There has been, as of yet, only a small number of publications in relation to the teaching and learning of mathematics in higher education during the COVID-19 pandemic although more are beginning to emerge. Several of these papers have focused on the education of prospective mathematics teachers and how the students adapted to the use of digital resources for mathematics (Mulenga & Marbán, 2020a, 2020b; Naidoo, 2020). A number of articles suggest alternative approaches to assessment in mathematics that are appropriate for remote teaching (Iannone, 2020; Jungic, 2020; Videnovic, 2020; Seaton, 2020). Other reports detail how mathematics support in higher education adapted during the emergency remote teaching (Hodds, 2020; Johns & Mills, 2021), highlighting in particular the ‘drastically reduced’ numbers engaging with the services during this time. Others consider how the community of mathematics post-secondary educators can learn from their experience of this time (Garaschuk & Jungic, 2020). An excellent resource for mathematics lecturers has been created in the UK (www.talmo.uk), which provides community-led online workshops and resources to assist lecturers with their practice.

Prior to the COVID pandemic, a growing body of research was emerging regarding the use of technology in mathematics lecturing in higher education (Trenholm *et al.*, 2012; Juan *et al.*, 2011; Oates, 2011), including areas such as the use of mathematics-specific software (Zambak & Tyminski, 2020; Hernández *et al.*, 2020; Caglayan, 2018), investigations into student and lecturer preference/usage of various mathematical resources (Ní Shé *et al.*, 2017; Inglis *et al.*, 2011) and explorations of approaches to mathematical problem-solving using technology (Abramovich, 2014; Kay & Kletskin, 2012). The question of providing recorded versions of live mathematics lectures for students has been explored for over a decade at this point (Cascaval *et al.*, 2008; Yoon & Sneddon, 2011) and is of particular relevance in relation to the stark choices that academics faced during emergency remote teaching due to COVID. In a study in which students had the choice of attending lectures or watching video resources (or both) in a first-year business mathematics module in Ireland, the majority chose the video resources, although those with higher lecture attendance in general received higher marks in the module (Howard *et al.*, 2018). Another study which considered students from five different mathematics modules in which both live lectures and video recordings were provided found that those who attended lectures in person considered video recordings of the lectures to be inferior to the live version (Yoon *et al.*, 2014), although lecture attendance was about 35% of the total cohort. Similarly, in a study comparing students in the UK and Australia, it was found that lower lecture attendance rates coupled with higher usage of video resources resulted in greater surface learning (Trenholm *et al.*, 2019).

Oftentimes, higher education lecturers reject the use of technology as unnecessary to their teaching until some powerful new incentive appears (Englund *et al.*, 2017). At this point, training and support for those adopting new technology, as well as positive initial experiences, are of paramount importance (Heinonen *et al.*, 2019; Jääskelä *et al.*, 2017). Prior research has shown that mathematics lecturers may not even be aware of the choice of additional hardware (e.g. visualizers, audio recording devices) accessible to them for teaching purposes within their own university, and there is a need for greater communication of such resources (Wood *et al.*, 2011). In addition, there is some evidence to suggest that mathematics lecturers are particularly wedded to their traditional form of lecturing (Sfard, 2014) and have been slow to embrace online teaching (Lokken & Mullins, 2014), with Engelbrecht & Harding (2005) questioning whether this was even possible in a discipline like mathematics. Maclaren (2014) postulates that the reluctance of mathematics lecturers to embrace newer technologies in place of a physical blackboard/whiteboard may be related to their perception that the trade-off is not worthwhile. Indeed, even the standard qwerty keyboard is tailored for text-based disciplines but does not easily translate into usage for representations of mathematics in an online environment (Trenholm & Peschke, 2020). Emergency remote teaching forced lecturers to adapt at short notice to teaching online, and so we explored what they used and for what purpose when they did so.

## 3 Materials and methods

In order to address the three research questions highlighted above, we designed and implemented a survey for mathematics lecturers in higher education.

### 3.1. Survey instrument

The online survey instrument began with a series of profile questions to determine the age profile, gender, country in which the respondent currently worked, years of experience teaching mathematics in higher education and current employment status. Following further profiling questions about class sizes, contact teaching hours and modules taught, there were six other sections in the survey (for the sections on assessment, see Fitzmaurice & Ní Fhloinn, 2021; the students’ experience, see Ní Fhloinn & Fitzmaurice , 2021a; the lecturers’ experience, see Ní Fhloinn & Fitzmaurice, 2021b; and advice for other practitioners, see Ní Fhloinn & Fitzmaurice, 2021c). In this paper, we focus upon two sections of the survey: types of technology and purpose of technology. Within these two sections, there were 29 questions of which 8 were open ended. The relevant questions are to be found in the Appendix.

**Table 1:** *Profile statistics of survey respondents (N =* 257*), showing their gender, age, years of experience teaching mathematics in higher education and current employment status*

	Number	**%**
Gender		
Male	135	52.5%
Female	118	45.9%
Non-binary	1	0.4%
No response	3	0.8%
Age		
20–29 years	16	6.2%
30–39 years	61	23.7%
40–49 years	69	26.8%
50–59 years	71	27.6%
60+ years	38	14.8%
No response	2	0.8%
Experience teaching mathematics in higher education		
0–1 year	8	3.1%
2–3 years	13	5.1%
3–5 years	20	7.8%
5–10 years	34	13.2%
10–15 years	31	12.1%
15–20 years	34	13.2%
20+ years	117	45.5%
Employment Status		
PhD/Postdoc	3	1.2%
Short-term contract (<=1 yr)	16	6.2%
Long-term contract (>1 yr)	28	10.9%
Permanent	205	79.8%
Retired but teaching	4	1.6%
No response	1	0.4%

### 3.2. Data collection

Ethical approval to conduct the study was granted by one of the author’s local ethics committee. The survey was conducted exclusively online using Google Forms and was emailed to mathematics department mailing lists and advertised via online conferences in mathematics education.

### 3.3. Data analyses

The quantitative data were analysed using Excel. General inductive analysis (Thomas, 2006) was employed to code the qualitative data. Coding was undertaken by both researchers independently. The codes were then compared and re-coded where necessary to ensure reliability. Throughout this paper, *N* is used to report the total number of respondents to a given question, while *n* is used for the number who selected a certain answer for a given question.

### 3.4. Sample

A summary of the profiling questions can be seen in Tables 1 and 2. There were 257 respondents. There was a fairly even breakdown of gender among the respondents, which would not be typical of surveys of mathematicians, given the fact that there are more male academic mathematicians than female. However, the survey was specifically emailed to a mailing list for female mathematicians, which contributed to the higher proportion of females answering the survey. The age of respondents showed a good spread with lower proportions under the age of thirty as would be expected among the lecturing population, and their years of experience teaching mathematics in higher education reflected this. The vast majority of respondents were in permanent employment.

By far, the highest proportion of respondents was based in Ireland, which is where both researchers are also based, and almost all respondents were based in Europe at the time of answering. However, in total, respondents from 29 different countries took part in the survey. These are displayed in Table 2. The countries in which only a single respondent was based are the following: Austria, Czech Republic, Denmark, Faroe Islands, Malta, Republic of Moldova, Sweden and Switzerland are listed collectively in the table (Other). Six respondents did not provide a base country (Not provided). Note that otherwise the table is a compilation of the exact responses given by respondents; for example, we have not collated England, Scotland, Wales and Northern Ireland with the general heading of UK (region not specified) as the education systems are not identical in each of these four countries.

**Table 2:** *Countries in which survey respondents (N =* 257*) currently work*

Country	Number	%
Ireland	78	30.4%
UK (region not specified)	37	14.4%
Germany	21	8.2%
Hungary	21	8.2%
England	17	6.6%
USA	12	4.7%
Scotland	10	3.9%
France	6	2.3%
Australia	5	1.9%
Italy	5	1.9%
The Netherlands	5	1.9%
Turkey	5	1.9%
Albania	3	1.2%
Slovakia	3	1.2%
Wales	3	1.2%
North Macedonia	2	0.8%
Northern Ireland	2	0.8%
Portugal	2	0.8%
Serbia	2	0.8%
Slovenia	2	0.8%
Spain	2	0.8%
Other	8	3.1%
Not provided	6	2.3%

The subjects being taught by the respondents could influence their approach to online teaching or their experience of this, particularly in terms of assessment (Iannone & Simpson, 2011), or whether they were teaching students specializing in mathematics (who would be studying many mathematics modules online) or students taking only a single mathematics module. Respondents were asked to list all courses they were teaching. Almost three-fifths of respondents were teaching students who were majoring in mathematics, with just over half of respondents teaching service-mathematics students (those studying one or more mathematics modules as part of their degree programme but not specializing in mathematics).

**Fig. 1.**
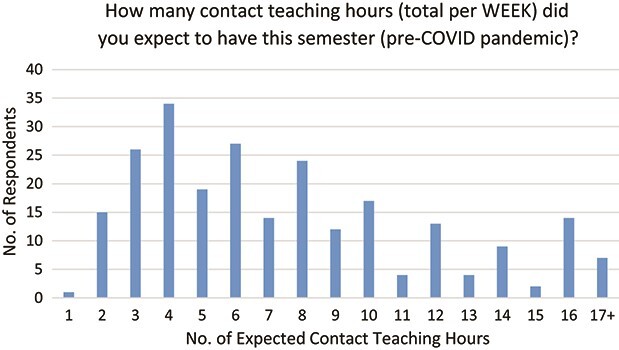
Number of contact teaching hours per week that respondents expected to have (pre-COVID pandemic) (*N* = 247).

The number of scheduled contact teaching hours that respondents had planned to do per week, pre-pandemic, was of interest as it would give a rough indication of the teaching workload involved. Figure 1 shows this for the 247 responses, with the vast majority of those teaching 8 hours a week or fewer.

Finally, the class sizes involved could impact upon a lecturer’s approach to emergency remote teaching and so respondents were asked to indicate the class sizes with which they were dealing with small being up to 30, medium being 30 to 100 and large being over 100. Of the 256 responses, 59.4% had small classes, 50.4% had medium classes and 22.7% had large classes.

### 3.5. Limitations of the study

(1) The current country of employment of 93% of respondents is somewhere in Europe with 30% of respondents in Ireland, meaning that the results may not be generalizable to other continents.
(2) The survey was only available in English and was conducted online, advertised via mailing lists and online conferences. There is no way of knowing how representative a sample it is of mathematics lecturers.
(3) The survey was undertaken during May/June 2020. At this point, some universities may not have completed their semester or examinations, which would impact some of the answers.
(4) Many respondents were in different stages in their academic year depending on the country in which they were located.

## 4 Results: hardware and software used by mathematics lecturers

To gain an insight into what hardware and software mathematics lecturers used during emergency remote teaching due to the COVID-19 pandemic, it was firstly important to ascertain what they used prior to this. As such, respondents to the survey were initially asked about the hardware/software they used prior to the pandemic and for what purposes, before then being asked the same question but for during emergency remote teaching. The full results are presented separately for hardware and software, to enable an easier comparison between the two time periods involved.

### 4.1. Hardware used before and during emergency remote teaching

**Fig. 2.**
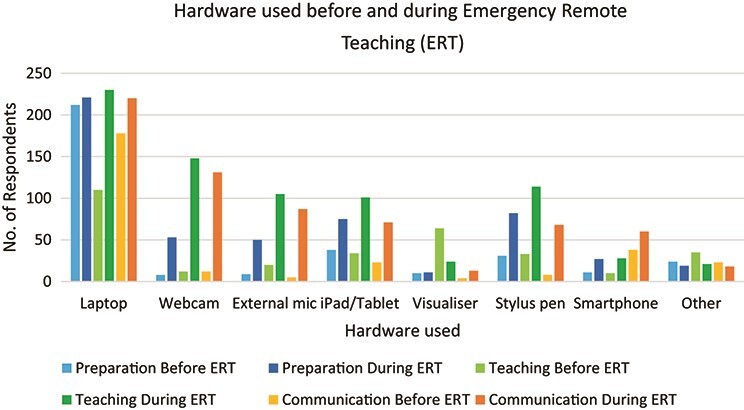
Types of hardware used before the pandemic/during ERT and their purposes (*N* = 257).

Figure 2 shows the responses to the questions ‘What types of hardware did you use in the workplace before the pandemic/during emergency remote teaching?’. Respondents were asked to indicate for what purposes they used the hardware in question, with three options available to them: for preparing materials, for teaching and for communicating with students.

It can be seen from Fig. 2 that there was very little use of different types of hardware prior to the pandemic, with almost all respondents indicating use of a laptop and only one quarter of respondents also using a visualizer (or document camera) for teaching purposes. By contrast, as might have been expected, a far wider variety of hardware was utilized by those involved in emergency remote teaching. Laptop usage was almost universal, with high usage of webcams, external microphones and stylus pens also reported. Visualizer usage for teaching had decreased, most likely due to lack of access.

In terms of the ‘other’ hardware used in various situations, the most common response (*n* = 29) was the use of a PC instead of a laptop, followed by a blackboard/whiteboard (*n* = 15). The other responses mostly would have been encapsulated in those shown in the figure or else were mentioned by only a couple of respondents.

### 4.2. Software used before and during emergency remote teaching

**Fig. 3.**
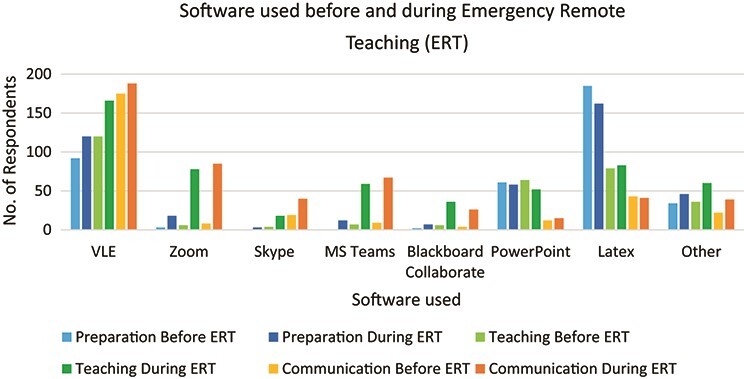
Types of software used before the pandemic/during ERT and their purposes (*N* = 257).

Having established what hardware was in use, respondents were then asked about their use of software both before the pandemic and during emergency remote teaching. Again, they were also asked for what purposes they used this software. The results are shown in Fig. 3.

The virtual learning environment (VLE) in their university was in use for all three purposes prior to the pandemic, with LaTeX also featuring strongly in the preparation of materials. PowerPoint also featured for both preparation and teaching purposes. During emergency remote teaching, the list became more diverse with communication systems such as Zoom, Skype, MS Teams or Blackboard Collaborate showing a wider usage.

In terms of ‘other’ software used, there were 101 different software listed by respondents, but 87 of these were mentioned by four respondents or fewer. The others are shown in Table 3 below.

**Table 3:** ‘Other’ software options which more than four respondents mentioned using either before the pandemic or during emergency remote teaching

Software	# Respondents
Cisco WebEx	9
OneNote	9
Big Blue Button	8
Email	8
Mathematica	7
OBS	7
Screencast-o-matic	7
YouTube	7
Geogebra	6
Canvas	5
Maple	5
Matlab	5
Panopto	5
MS Word	5

### 4.3. Selection of technology

Respondents were asked a general question regarding who chose the technology that they used and were allowed to select as many options as were applicable: 81.3% (*n* = 208) of respondents said they chose it themselves, 21.5% (*n* = 55) said decisions were made at departmental level, while 53.1% (*n* = 136) said it was at university level. From comments made throughout the survey, this would seem to indicate that the department/university chose what paid technologies to make available, and most lecturers were then in a position to choose which technology they would use from what was available.

## 5 Results: remote teaching practices

**Fig. 4.**
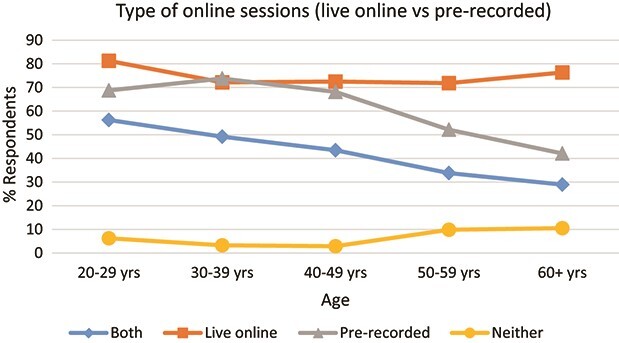
Types of online sessions (live online/pre-recorded/both/neither) run by respondents during ERT, subdivided by age-group (*N* = 255).

Having looked in Section 4 at a macro level at the ways in which the hardware and software were used by mathematics lecturers, both before the pandemic and during emergency remote teaching, we then focussed on uncovering the specific forms of teaching mathematics lecturers pursued during the latter. Almost three-quarters of respondents (*n* = 189) gave some form of live online sessions, and over three-fifths produced pre-recorded sessions (*n* = 156). Just over two-fifths undertook both live online sessions and pre-recorded sessions (*n* = 104). There were no significant differences between male and female lecturers in terms of which form of online teaching they undertook, but some differences were observable between lecturers in varying age groups, as shown in Fig. 4. In general, lecturers were less likely to use pre-recorded sessions as they got older and slightly more likely to do neither live online nor pre-recorded sessions. It should be remembered, however, that there were smaller numbers of the 20–29 years and 60+ years cohorts within the overall sample, and so these results must be interpreted with this in mind.

**Fig. 5.**
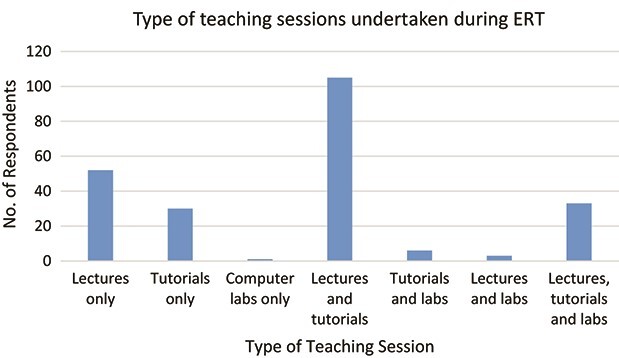
Types of teaching sessions (lectures/tutorials/computer labs) conducted by respondents during ERT (*N* = 256).

About three-quarters of respondents conducted lectures online (*n =* 193), almost 68% gave online tutorials (*n* = 174) and nearly 17% did online computing labs (*n* = 43). Figure 5 shows the breakdown of what combinations of teaching sessions were undertaken. There were no significant differences between lecturers of different genders or age groups in this case.

In order to further explore the reasons for choosing to give live sessions versus pre-recording material, respondents were asked to comment further on these issues, as reported below.

### 5.1. Live sessions

**Fig. 6.**
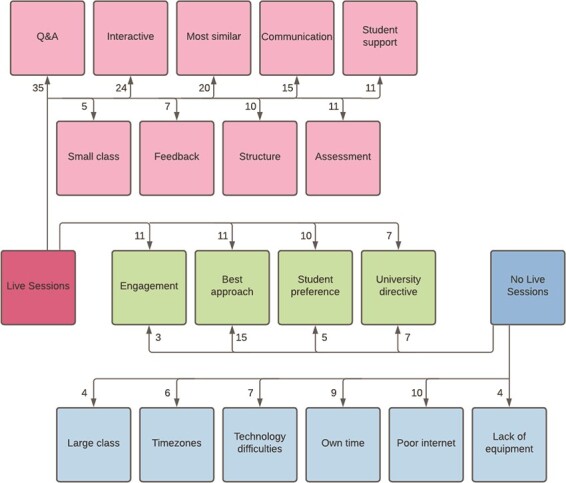
Themes which emerged from the question: ‘Did you give live sessions online and why/why not?’ The number of comments under each theme are indicated by the numbers present on the arrows.

A total of 203 of the 257 who responded about whether or not they gave live online sessions commented further to explain why. Of these 203 commenters, 71.4% had given live sessions. The comments were split into those made by respondents who conducted live sessions, and those made by those who did not, as shown in Fig. 6. Comments were coded under more than one theme, where applicable.

The most common theme among those who had conducted live online sessions was that of facilitating ‘Question and Answer’ sessions (*n =* 35, 17%) with most highlighting the importance they placed upon *‘giving students the opportunity to ask questions’.* Closely linked to this was the lecturers’ desire to make sessions ‘interactive’ (*n* = 24, 12%) (*‘I believe that interactions with students are essential’*) and their overall feeling that offering live sessions was the ‘most similar’ to what students were used to (*n* = 20, 10%) (*‘All sessions were live as I felt it was best to try and normalize the experience as much as possible’*). Many also mentioned that ‘communication’ was an important part of their teaching (*n* = 15, 7%) (*‘personal communication is absolutely necessary to teach the type of material I taught for undergrads’*) and that this was facilitated by live sessions. Another theme to emerge was that of ‘student support’ (*n* = 11, 5%), with respondents feeling that *‘the psychological impact of live sessions helped my students greatly’*. The theme of ‘assessment’ emerged also (*n* = 11, 5%) with respondents using live sessions to conduct student presentations (*‘in this course the students are supposed to present their seminars’*), group-work assignments or project supervision. Some felt strongly that regular live sessions were useful in providing a ‘structure’ with which students were familiar (*n* = 10, 5%) (*‘I felt it would help students stay motivated and structure their time if there was still a schedule to follow’*). Finally, lecturers found live sessions important *‘to get direct feedback from the students’* (*n* = 7, 3%) but also acknowledged that they were particularly suitable for a ‘small class’ group (*n* = 5, 2%) (*‘Had a small Masters class. So Teams meeting seemed the best way to go. For my other module, there were 180 students. Felt that was too big for the meeting format’*).

There were four common themes across those who had and had not given live sessions: for example, respondents from both groups mentioned being given a ‘university directive’ to either give live online sessions or not to (live: *n* = 7, 3%; not live: *n* = 7, 3%). Similarly, ‘student preference’ was a deciding factor for both groups (live: *n* = 10, 5%; not live: *n* = 5, 2%). In terms of ‘engagement’ (live: *n* = 11, 5%; not live: *n* = 3, 1%), those who did live sessions *‘felt it was very important, both academically and from a well-being perspective, to keep the students engaged’*, while those who did not avoided them due to *‘uncertainty about participation rate’*. The most frequent common theme between both groups was that of their approach being the ‘best approach’ (live: *n* = 11, 5%; not live: *n* = 15, 7%), with those who did live sessions stating *‘this was the best thing given the nature of some of my teaching’* and those who did not feeling that there was not ‘*any great pedagogical advantage versus recorded lectures’.*

There were six other themes identified among the responses of those who did not offer pre-recorded sessions. Most prominent among these was ‘poor internet’ (*n* = 10, 5%), with lecturers concerned about the low quality of their own internet connection or that of their students. Other respondents felt that it was important to allow students to engage with material in their ‘own time’ (*n* = 9, 4%), rather than having scheduled sessions (*‘I thought best…that students could participate when it suited them best. This was to try to reduce any pressure on them’*). Some lecturers spoke of ‘technology difficulties’ that prohibited them from conducting live sessions (*n* = 7, 3%) (‘*the technology (tablet+mic+camera) adds sufficient extra difficulty that I do not think it would be manageable’*), while others mentioned the fact that their students live in different ‘time zones’ *‘who would not have been able to tune into the live sessions’* (*n* = 6, 3%)*.* Finally, ‘large class’ sizes dissuaded some from live online sessions (*n* = 4, 2%) (*‘class is too large for live session (400 students)’*), along with ‘lack of equipment’ (*n* = 4, 2%), particularly during the initial weeks (*‘I did not have a visualizer to show students calculations in real time. I did not have a tablet for several weeks’*).

### 5.2. Pre-recorded sessions

**Fig. 7.**
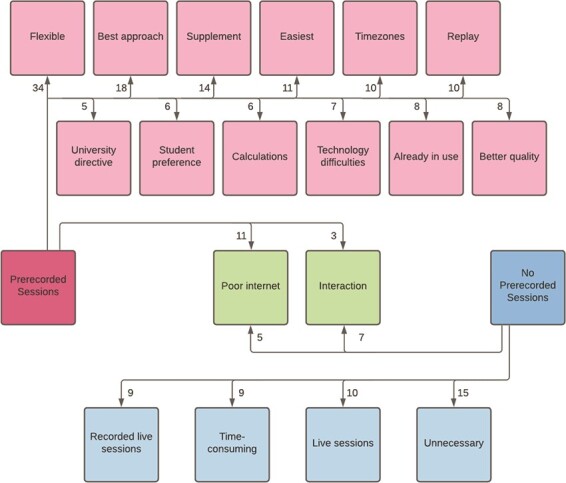
Themes which emerged from the question: ‘Did you do any pre-recorded sessions and why/why not?’ The number of comments under each theme are indicated by the numbers present on the arrows.

A total of 201 of the 254 who responded about whether or not they did pre-recorded sessions commented further to explain why. As above, the comments were split into those who had conducted pre-recorded sessions and those who had not, as shown in Fig. 7. Again, comments were coded under more than one theme, where applicable**.**

A large number of themes emerged from those who pre-recorded material. Chief among these was the ‘flexible’ nature of such content (*n* = 34, 17%), both for students (*‘Allowed students to work when suited them’*) and lecturers themselves (*‘since I am at home with my family, it was easier to work at any particular time, rather than following the school’s timetable’*). The next most common theme was that of pre-recorded sessions being the ‘best approach’ under the circumstances (*n* = 18, 9%) (*‘It seemed like the best available substitute for live lectures’*), closely followed by the fact that others intended these pre-recorded sessions as a ‘supplement’ for other approaches (*n* = 14, 7%) (*‘For higher level modules, where additional materials are not easily available online from other sources, I though it is of benefit to offer short focussed videos on key concepts’*). Other respondents found pre-recorded sessions to be the ‘easiest’ approach (*n* = 11, 5%) (*‘Since I have an iPad and my institution already subscribes to Explain Everything, recording pencasts was very easy to do and was an obvious tool to use’*), with others mentioning the ability for students to ‘replay’ the material as a clear advantage (*n* = 10, 5%) (*‘Students can pause and replay if they do not get the idea at once’*). Different ‘time zones’ emerged as a theme for a number of respondents (*n* = 10, 5%), with pre-recorded sessions allowing them *‘to address students being in multiple time zones’.* For some respondents, pre-recorded sessions ensured that their teaching was of ‘better quality’ (*n* = 8, 4%) (*‘I would demand of myself that such videos were more polished than a livestream. Even though those are also recorded’*), while for others, this approach was ‘already in use’ (*n* = 8, 4%) (*‘I have always used short podcasts’*) and as such, it made sense to continue to incorporate these during remote teaching. Fear of ‘technical difficulties’ during live sessions drove some towards pre-recording (*n* = 7, 3%) (*‘Technical issues made me fear that I couldn’t do anything reliably at the beginning’*), while others reported that it was the ‘student preference’ (*n* = 6, 3%) (*‘I had some positive feedback from students about having both the video and the annotated slides from the end of the video. They could look over the annotated slide to see if they understand what was done in the video, and then watch the video, knowing that they might need to pay special attention to some parts, or be able to skip over parts that already make sense to them*’). The sense that students *‘need to see calculations develop’* was put forward as another reason for pre-recorded sessions (*n* = 6, 3%), while other respondents stated that it was a university directive (*n* = 5, 2%), either because *‘initially, the university could not provide services for live sessions’* or they *‘preferred us to upload recorded classes instead of performing live, so as not to overload the university server’.*

There were only two common themes between those who pre-recorded sessions and those who did not: ‘poor internet’ (pre-recorded: *n* = 11, 5%; no pre-recorded: *n =* 5, 2%) and ‘interaction’ (pre-recorded: *n* = 3, 1%; no pre-recorded: *n =* 7, 3%). In terms of those who pre-recorded sessions, most of those who mentioned internet issues were *‘concerned that poor broadband would negatively impact on student engagement with live lectures’* while those who did not pre-record were either unable to upload recordings due to their own poor internet speed or worried that students would not be able to download them for similar reasons. In terms of interaction, those who pre-recorded mentioned low interaction levels in lectures, so they felt there would be little lost by simply pre-recording while those who did not pre-record spoke of how they ‘*believe that interactions with students are essential’* and felt pre-recording would not allow for this.

There were four other themes identified among the responses of those who did not do pre-recorded sessions. The most common of these was that respondents felt they were ‘unnecessary’ (*n* = 15, 7%), and they did not comment further on this. Several respondents mentioned preferring to do ‘live sessions’ instead (*n* = 10, 5%) (*‘Live sessions seemed to me better since that way I could speak directly with students and answer to their questions and preoccupations’*), while others specifically referenced ‘recording live sessions’ as an alternative approach (*n* = 9, 4%) *(‘Live sessions were recorded, which (in part) did away with the need for pre-recording*’). Finally, the ‘time-consuming’ nature of pre-recording also emerged as a theme (*n* = 9, 4%) (*‘Generating additional pre-recorded material would have required significant additional time’*).

### 5.3. Alternative approaches

From those who did not offer lectures, tutorials or computing labs online, there were a number of alternative approaches taken, with 32 respondents providing feedback on this. The most common approach (*n* = 11, 34%) taken was to conduct online assessment or give students exercises to complete and submit for feedback. Next most popular (*n =* 10, 31%) was to help students on an individual basis, either through email or video consultation sessions. Another popular approach (*n =* 9, 28%) was to provide videos for the students, made available through their VLE, YouTube or directly emailed to students. Nine respondents (28%) provided lecture notes, which they sent to their students for self-study, though a couple mentioned that they only took this approach because their module was almost completed and there was little content remaining to be covered. Finally, a small number (*n =* 4, 12%) of respondents set up discussion forums for student use.

## 6 Results: training and support

### 6.1. Training

Respondents were asked about the formal training they received in the use of any technology. 44.9% of respondents (*n* = 115) stated that they were offered formal training in the use of technology by their employer prior to the pandemic, with 54% (*n* = 62) of these availing of such training. Once the pandemic began, 65% of respondents (*n* = 166) were offered formal training, and 45% (*n* = 74) participated in this. Of this pandemic-related training, 10.3% (*n* = 12) stated that the training took place at the outset of the pandemic just prior to emergency remote teaching, 56% (*n* = 65) stated it took place during emergency remote teaching, with 33.6% (*n* = 39) stating training was available both before and during emergency remote teaching.

For those who did not give tutorials and/or computing labs online themselves, 53% had a teaching assistant do so instead (*N* = 103). In terms of training these teaching assistants in the use of the necessary software, the responsibility for this lay in a variety of areas: 35% (*n =* 22) of respondents said it was their responsibility as lecturer, a further 32% (*n =* 20) said it was the teaching assistant’s own responsibility, while 19% (*n =* 12) stated it was up to the university and 14% (*n =* 9) that it was up to their department.

### 6.2. Technical support

When asked if they had access to technical support if needed during emergency remote teaching, 92.5% (*n* = 234) of respondents stated that they did, with 5.9% (*n* = 15) having no such access. However, numerous comments under this question stated that the technical support was overwhelmed at this point in time (*‘In theory yes, but have received no help. Staff were either overloaded and could not help or just weren’t able to do what I requested*’). A total of 55.1% (*n* = 141) of respondents stated that they experienced technical difficulties during online teaching that took place during the early period of the COVID-19 pandemic. The most common technical difficulties encountered at this time were poor internet connection (*n* = 67, 26%), software issues (*n =* 60, 24%), hardware problems (*n* = 30, 12%), communication difficulties (*n* = 22, 9%) and technical difficulties for students (*n* = 22, 9%). Poor internet connections were generally mentioned in relation to issues providing live online classes (‘*very poor internet connection from my house…making it impossible to run live lectures*’). Software issues ranged from ‘*trouble with installation of programmes*’, to ‘*unfamiliarity with software - needed to learn how to master it*’, to software errors, particularly in relation to assessments (‘*issues with online assessment of maths via BrightSpace—no records being sent to the GradeCentre*’), and finally, software deemed not fit for purpose (‘*I followed the advice of the university on what software and hardware to use to teach a highly-interactive class. The advice was only theoretically good. In practice, the proposed combination is not workable*’). Hardware problems are usually related to old equipment (*‘very old laptop that I have to have an icepack under else it overheats’*), lack of equipment (‘*no printer/scanner available in home office’*) or unsuitable equipment (‘*the external webcam does not work well to shoot on my handwriting*’). Communication difficulties often referred to problems with Zoom/Teams/Adobe Connect (‘*issues with Zoom using dual monitors with different resolutions*’), although a few respondents also had difficulties with their email system (‘*earlier I did not use my University mail system, it was difficult to get passwords etc.*’). Finally, technical difficulties for students resulted from ‘s*tudents without access to stable internet*’ or lack of hardware/software to participate as needed in the class.

When asked to list all those who resolved these difficulties for them, there were 139 responses of which 53% said they resolved their difficulties themselves, 26% stated it was technical support, 25% with issues still outstanding and 9% had help from colleagues or family members.

## 7 Discussion and conclusion

In this paper, we posed three main research questions. The first of these is related to the hardware and software used by mathematics lecturers both before the pandemic and during emergency remote teaching. Over four-fifths of lecturers were in a position to choose the hardware/software they used themselves, allowing them a large degree of autonomy in this regard, although they were usually making their selection from a pre-determined menu of options in relation to anything that was not free to use. Prior to the pandemic, there was low usage of different types of hardware, with laptops the only exception to this, with 88% of respondents using these for some purpose (additionally, it should be noted that some respondents reported using PCs instead).Visualizers, stylus pens and smartphones saw some low levels of usage for particular purposes with 27%, 17% and 18% usage, respectively, although the low usage could have been due to lecturers’ lack of knowledge of how to access these, as was the case in Wood *et al.* (2011). In fact, only two-fifths of respondents reported using laptops for teaching purposes and a third did not report using any form of hardware for teaching purposes, with many of these relying instead on physical blackboards/whiteboards (*‘Before the closure, I used a chalkboard in all of my classes’*)*.* This echoes the findings of Billman *et al.* (2018), who found in their study of mathematics lecturers in a South African university that *‘(d)espite this remarkable increase in technology usage for teaching, half of the teaching staff still prefers the use of a chalkboard to technology for teaching’.* Indeed Greiffenhagen (2014) argues that the blackboard has an ‘almost iconic stance’ in mathematics, due to the nature of mathematics and the importance lecturers attach to the visibility of the ‘process of mathematical reasoning’ it provides. This is backed up in our study by the observation that, among our respondents, there was little discernible difference between age groups or experience levels in terms of their reliance upon physical blackboards, with between 10% and 14% of 30–39, 40–49 and 50–59 year olds explicitly mentioning their use of physical blackboards at various points during the survey and 6% and 5%, respectively, of 20–29 year olds and 60+ year olds.

During emergency remote teaching as might be expected the types of hardware in use both diversified and increased considerably, as well as the range of functions for which the hardware was being used. The percentage of respondents reporting no hardware usage for teaching reduced from a third to 3.5%. However, respondents reported difficulties accessing hardware at short notice, with numerous cases of lecturers resorting to borrowing (*‘I needed a tablet and pen but the shops were closed. I borrowed’*) or relying on personally owned equipment in their home (‘*Had I not had a number of devices at home, personally bought, then I would not have been able to perform my role as successfully as I had’*). Respondents in many cases aimed to replicate the experience of having access to a physical blackboard/whiteboard, the format most familiar and comfortable to a large number of mathematics lecturers (Artemeva & Fox, 2011). Despite this, the number using visualizers or even make-shift visualizers based on adapting their smartphones (e.g. University of Oxford, Mathematical Institute, 2020) remained surprisingly low, suggesting that lecturers may not have been aware of this possibility.

In relation to software, the results were similarly sparse before the pandemic with 76% of respondents using their institutional VLE for some purpose, 74% using LaTeX and 28% using PowerPoint. Here, the low numbers using PowerPoint may be as a result of its perceived ineffectiveness for teaching mathematics, between the difficulty of representing mathematics on the slides and the static nature of this mode of presenting (Loch & Donovan, 2006). Indeed during emergency remote teaching, the numbers using PowerPoint for teaching purposes actually decreased slightly to 26%, showing it was not a perceived solution during emergency remote teaching to online teaching in mathematics. The number using their institutional VLE increased slightly to 79% during emergency remote teaching. However, it has been observed that VLE usage in those moving initially to online teaching can simply be an attempt to create a digital version of an existing paper-based resource, without any further alteration for the online environment (Borba *et al.*, 2016). Our figures show that lecturers were more likely to use their VLE for communication purposes with students rather than for teaching purposes, suggesting that while they were making use of the various communication tools within the VLE, they were less inclined to use (or less familiar with) the teaching technologies therein. Large increases were reported during emergency remote teaching in communication software usage as might have been expected with 39% now using Zoom, 29% using Teams, 17% using Skype and 14% using Blackboard Collaborate. There was a far greater diversity in the range of ‘other’ software used by respondents but no strong trends towards particular software emerged. A total of 18% of respondents used some ‘other’ software before the pandemic and this increased to 28% during emergency remote teaching, but respondents encountered a range of difficulties in implementing and adapting to new software: ‘*There was a VERY steep learning curve in obtaining the necessary software, learning how to use it and getting the laptop connected to successfully continue with the classes’* and ‘*One software allows you up to so many users but your class is too big, the other has issues with security, the third is better for recording but not real-time teaching’*. This feedback suggests that many respondents did not have the ‘positive initial experience’ that Heinonen *et al.* (2019) identified as being so valuable when introducing new software for teaching.

Our second research question aimed to delve deeper into the purposes for which lecturers used this hardware and software by exploring why lecturers opted to conduct live online sessions, pre-recorded sessions or alternative approaches. The most common approach was to give live sessions online with pre-recorded sessions also a prominent approach, and many respondents doing both. Only a very small number (*n* = 8) of respondents mentioned having used pre-recorded sessions previously, and yet 156 developed pre-recorded sessions during this period, bearing out the observation of Englund *et al.* (2017) that a powerful new incentive can lead lecturers to use technology they would previously have considered unnecessary. The importance placed by lecturers on interactivity and the ability to ask and answer questions in real time emerged strongly in the responses, and this was reflected in student reasons given for their preferences for live lectures in the work of Yoon *et al.* (2014). Indeed, strong justifications were given by respondents both in favour of and against providing live online sessions, with similar responses for pre-recorded sessions. These responses mirror in many ways the opposing opinions expressed in relation to the recording of live in-person lectures, highlighted earlier (Howard *et al.*, 2018; Trenholm *et al.*, 2019; Yoon & Sneddon, 2011). At the time of the survey, the impact of one choice over another is not yet clear in this situation, where many lecturers and students were living in lockdown conditions for at least a portion of this time and where assessments also had to change at short notice. However, it is worth noting that respondents from both sides deemed their approach to reflect ‘student preference’. It should be observed that students do not always choose the most effective strategies for their own study (Bjork *et al.*, 2013) and ‘popular’ choices may not necessarily result in the most learning, as evidenced by the low correlation between student evaluations of teaching and evidence of learning (Uttl *et al.*, 2017). The observation made by Trenholm *et al.* (2019) about the higher level of surface learning associated with those who chose to engage only with video resources when live in-person lectures were available may not correspond to this cohort of students, given the particular circumstances in play, and the fact that students may have only had one option available in their module. Yet, engagement levels emerged as a strong concern among lecturers, regardless of the approach they took, with some observing that ‘*viewing figures for pre-recorded sessions was very poor’* and others that ‘*tutorials were offered by our tutors but not many availed of them*’ or that *‘I offered “class time” for answering questions etc. There was very little take-up’*. This echoes the experience of those involved in mathematics support at that time, who universally reported far lower engagement figures with their services during this period (Hodds, 2020). This lack of engagement with online teaching in mathematics is not unique to emergency remote teaching, with Glass & Sue (2008) reporting that, although students in their fully online mathematics module engaged well with homework, they placed little value on any discussion forums or similar. Many lecturers spoke of the benefits of the flexibility of pre-recording sessions, both for themselves but primarily for their students, some of whom were in different time zones or shouldering known and unknown responsibilities at the time. It should be noted that, across many of the themes that emerged in responses to questions regarding the purpose for which technology was used, a concern for their students and a desire to provide the best possible educational experience for them under the circumstances permeated many of the comments made by respondents, with one simply observing *‘I miss my students’*.

Our final research question focused on the training and support that lecturers received in any hardware or software they used. There was a steep learning curve involved, one which is often made more difficult in the case of mathematics-specific software by the different requirements of software packages when representing mathematical language (Smith *et al.*, 2008). Effective support of lecturers moving to a fully online environment has been hypothesized to rely on three components: administrative support, peer support and professional development (Covington *et al.*, 2005), and it was the last of these that we particularly investigated here. The percentage of respondents who were offered professional development opportunities in use of educational technology was relatively low, given the circumstances, with less than half reporting any training available prior to the pandemic and only two-thirds during emergency remote teaching. Given the importance of effective training and support to ensure lecturer engagement with new technologies of this kind (Jääskelä *et al.*, 2017; Heinonen *et al.*, 2019), it is vital for universities to support their teaching staff in this manner in the event of any sudden closure. It is also incumbent upon the lecturers to avail of such training as befits their needs, with less than half reporting engaging with the training offered by their university during emergency remote teaching. It would be of interest in the future to investigate their reasons for non-engagement: whether they were personal circumstances during emergency remote teaching or whether the training was not bespoke to technology relevant to mathematics and therefore perceived not to be of use. The vast majority of respondents reported having technical support available to them during this time period, even if it was, as might have been expected, often overwhelmed with the number of requests. It is worth noting however that at the time of this survey some 3–4 months after the beginning of emergency remote teaching, 25% of those who experienced technical difficulties had not yet had these resolved. This may, in part, have been due to the fact that many were still in a state of lockdown and there were difficulties with accessing in-person support with hardware or wifi provision but also points to a difficult transition to emergency remote teaching for a significant portion of respondents.

The results of this survey provide a snapshot into a unique period of emergency remote teaching, as mathematics lecturers adjusted their teaching practices, making use of whatever hardware and software were available to them and their students in the circumstances of emergency remote teaching. The creation of a student-centred environment which was both interactive and engaging was mentioned in some format by the majority of respondents. However, the tension between replicating a teaching and learning environment as close as possible to that with which they were most comfortable prior to emergency remote teaching, under conditions that were less than conducive to teaching and learning, was evident in the responses given. Adequate access to hardware/software, and professional development in the use of ICT to teach mathematics, will be a requirement in all universities, as it is likely in the future that there will be a move towards more online provision of teaching both for the duration of the COVID pandemic and beyond (Blankenberger & Williams 2020). We believe that it is simultaneously incumbent on universities to provide hardware, software and training and for staff to uptake and participate. Consideration of student access is critical.

Further research into best practice is also necessary. We plan to reissue our survey a year after the original survey was distributed to ascertain the developments within online teaching of mathematics during this time. A spontaneous move to remote teaching is a significantly different experience to online teaching with a year’s experience. A comparison of the initial starting point as reported in this paper, with the developments over the course of a year, should provide new insights into the extent of this difference.

## Notes

**Dr Eabhnat Ní Fhloinn** is an associate professor in the School of Mathematical Sciences and director of DCU Maths Learning Centre. Her research interests lie in the area of mathematics education, with particular focus on the transition from post-primary mathematics to higher education, mathematics support in higher education, mathematics diagnostic testing of incoming university students, mathematics for engineers and assessment in mathematics.

## Notes

**Dr Olivia Fitzmaurice** is a lecturer in Mathematics Education in the School of Education and academic director of the Mathematics Learning Centre at the University of Limerick. Her research focuses primarily on preservice mathematics teacher knowledge and the provision of effective mathematics learning support.

## Appendix: survey questions

### Profile Questions

The following are some general questions about you...

(1) Country in which you currently work
(2) Gender: Female/Male/Prefer not to say/Other
(3) Age: 20–29 years/30–39 years/40–49 years/50–59 years/60+ years
(4) Number of years experience teaching mathematics in higher education: 0–1 year/2–3 years/3–5 years/5–10 years/10–15 years/15–20 years/20+ years
(5) Status of your current role: Permanent/Long-term contracts (greater than 1 year)/Short-term contract (1 year or less)/Other

***Mathematics Teaching Allocation***

These questions ask some details about your teaching allocation for the semester just past.

(1) How many contact teaching hours (total per WEEK) did you expect to have this semester (pre-COVID pandemic)?
(2) Which of the following did you teach this semester? (Please tick all that apply): Students majoring in mathematics/Students taking non-specialist (service) mathematics/Algebra/Analysis/Discrete Maths/Geometry/History of Mathematics/Mathematics Education/Mechanics/Operations Research/Probability/Statistics/Other
(3) What were your class sizes? (Please tick all that apply): Small (up to 30)/Medium (30 to 100)/Large (greater than 100)

***Types of Technology***

This section explores what technology types you used

(1) What types of hardware did you use in the workplace before the pandemic?

**Table:**

	For preparing materials	For teaching	For communicating with students
Laptop			
Webcam			
External microphone			
iPad			
Tablet			
Visualiser			
Stylus pen			
Smartphone			
Other (please elaborate below)			

(2) What types of hardware did you use during the university closure?

**Table:**

	For preparing materials	For teaching	For communicating with students
Laptop			
Webcam			
External microphone			
iPad			
Tablet			
Visualiser			
Stylus pen			
Smartphone			
Other (please elaborate below)			

(3) What other hardware did you use (for prep/teaching/communication)?
(4) What types of software did you use in the workplace before the pandemic?

**Table:**

	For preparing materials	For teaching	For communicating with students
Virtual Learning Environment (VLE) (eg Moodle, Blackboard)			
Zoom			
Skype			
Microsoft Teams			
Bongo			
Adobe Connect			
Blackboard Collaborate			
Webworks			
Bitpaper			
PowerPoint			
Active Presenter			
Explain everything/Explain Edu			
Facebook groups			
LaTeX			
Other (please elaborate below)			

(5) What types of software did you use during the university closure?

**Table:**

	For preparing materials	For teaching	For communicating with students
Virtual Learning Environment (VLE) (eg Moodle, Blackboard)			
Zoom			
Skype			
Microsoft Teams			
Bongo			
Adobe Connect			
Blackboard Collaborate			
Webworks			
Bitpaper			
PowerPoint			
Active Presenter			
Explain everything/Explain Edu			
Facebook groups			
LaTeX			
Other (please elaborate below)			

(6) What other software did you use (for prep/teaching/communication)?
(7) Any further comments on your use of hardware/software?
(8) Who chose the technology that you used? Myself/Department/University/Other
(9) Prior to the COVID-19 pandemic, were you offered any formal training in the use of technology by your employer? Yes/No/Don’t Know
(10) If yes, did you avail of such training prior to the COVID-19 pandemic? Yes/No
(11) Once the COVID-19 pandemic began, were you offered any formal training in the use of technology by your employer? Yes/No/Don’t Know
(12) If yes, did you avail of formal training? Yes/No
(13) If yes, was this training before and/or during university closure? Before closure/During closure/Both before and during closure
(14) Did you have access to technical support if needed during university closure? Yes/No
(15) Did you experience technical difficulties during online teaching that took place during the COVID-19 pandemic? Yes/No
(16) If yes, what was the nature of these difficulties?
(17) If yes, who resolved these technical difficulties? Myself/A colleague/Technical support/Still unresolved

***Purpose of Technology***

This section looks at what kind of emergency remote teaching you were engaged in during the university closure.

(1) Did you do any live sessions? Yes/No.
(2) Why/why not?
(3) Did you do any pre-recorded sessions in any format?
(4) Why/why not?
(5) Did you give lectures online in some format? Yes/No
(6) Did you give tutorials online in some format? Yes/No
(7) Did you give computing labs online in some format? Yes/No
(8) If you did not give tutorials/computing labs online, did a tutor do so instead? Yes/No
(9) If yes, whose responsibility was it to train the tutor in the use of the software? Tutor’s Own/Mine/Department/University/Other
(10) If there were no lectures and/or no tutorials and/or no computing labs conducted in a module where there usually are, what did you do instead?
(11) Please provide any further details, not referenced above, of how you conducted lectures/tutorials/computing labs remotely
(12) How often did you need to conduct teaching of any description online? Several times a day/Once a day/Several times a week/Once a week/Several times a fortnight/Once a fortnight/Other
